# Notices

**Published:** 1866-12

**Authors:** 


### NOTICES.

Subscriptions to HALL'S JOURNAL OF HEALTH expire with the December Number. Those who do not wish to take it for another year, need give no notice, as it is never sent without an express order, accompanied with the subscription price.

**THE CHILDREN'S HOUR:** A NEW MAGAZINE FOR THE LITTLE ONES. Edited by T. S. Arthur.— Our new Magazine will come as a pleasant companion, friend and counsellor of the little ones; and as a helper in the work of storing up things good, and true, and beautiful in their minds, through a healthy culture of the imagination and an attractive illustration of those precepts that lie at the foundation of all right living. It will aim to inspire children with reverence for God and a sense of His loving and fatherly care; and to lead them to unselfish actions — to be gentle, forbearing, merciful, just, pure, brave, and peaceable. *Some of the best writers for children in the country will contribute to its pages.*

THE CHILDREN'S HOUR will be as elegant in appearance as the best artists and the best typography can make it. The first number will be ready on the first of November, and will be mailed as a sample on receipt of ten cents.

Terms: — One year, in advance, $1.25. Five copies, $5.00. Ten copies, $10.00, and an extra copy to the person sending the club.

For $3 we will send one copy of the Home Magazine and one copy of the Children's Hour.

Address T. S. ARTHUR, 323 Walnut St., Philadelphia.

Mr. Godey, in his Lady's Book, thus speaks in advance of this new enterprise:

T. S. ARTHUR'S NEW MAGAZINE FOR CHILDREN.—We take more than usual pleasure in referring our readers to the prospectus of THE CHILDREN'S HOUR, a new magazine for the Little Ones. No one in the country is more widely or favorably known as a writer for children than Mr. Arthur, and thousands of mothers who enjoyed and profited by his beautiful story-lessons, when young, will gladly accept the opportunity of placing this new magazine in the hands of their children.

We understand that Mr. Arthur has long contemplated issuing a magazine for the young, but other literary engagements drew so heavily upon his time and health that he could not at any earlier period commence its publication. Now, all things favor the undertaking, and he comes to it with a loving interest in the work, delayed for years, that must insure its excellence and success.

"THE CHILDREN'S HOUR" will not be the rival of any other juvenile periodical, but have its own distinctive features, and address itself to the work of helping the little ones to take the first steps in life safely and pleasantly, in its own peculiar way. It will be the mother's assistant, as well as the child's companion, friend and counsellor.

*Don't fail to get a number.* We hope that every mother who takes the Lady's Book will take Mr. Arthur's Child's Magazine also."

HALL'S JOURNAL OF HEALTH, which is published monthly at $1.50 a year, will be sent, with *The Children's Hour*, during 1867 for $2.00, if sent previous to December 31st., 1866, to "Hall's Journal of Health, No. 2 West 43d St., New York."

The American Tract Society, 28 Cornhill, Boston, and 13 Bible House, New York, corner of 8th St. and 4th Avenue, have issued for the Sunday reading of families, "There's Time Enough ;. or, The Story of Charles Scott." 153 pps. "Our Charley ; or, The Little Teacher." Pps. 125. "Winnie and Her Grandfather ; or, the way to overcome evil with good." 144 pps. "Made Graves :" by the author of Jessie Lovell. "Grace's Visit ;" A Tale for the Young. 247 pp. All of these are safe and instructive reading for the young, which with a multitude of other books, as useful and as good.

Messrs. Broughton and Wyman will be happy to sell at low prices to all who pay them a visit at 13 Bible House, New York.

### TO BAPTISTS.

It is well known that the Watchman and Reflector, published weekly at Boston, Mass., at $3 per year—if paid within three months—has now been published forty-seven years, and has been edited with a steady ability and intelligence not surpassed by any paper in the Baptist Church. In one important respect it has been faithful above all others in guarding its readers against the patronage of what are considered respectable publications and periodicals, but which, every once in a while, were found covertly spitting out their venom and infidelity against the Christian Religion, thus being a watchman wide awake and independent and fearless enough to attack in high placce. Too many religious publications praise indiscriminately all that comes from publishing houses which send them a great many books and give them a large advertising patronage ; this is their shame.

The Boston Watchman and Reflector has not been with this crowd of sleepy watchmen, and we trust it never will.

To give some idea of the value of the reading in a single number, we take up the first at hand, for October 25, 1866.— A letter from London, from Peter Bayne. Inspiration of the Scriptures. The Savior's Invitation Accepted. Encouraging Inquirers. A Greeting with Paul. Letters from England and Ireland—and all this on the first page, the three others being filled with valuable practical matter. No family of any denomination could possibly read such a paper a year without being the better for it. The reading is uniformly of such a character as to elevate and purify and instruct wherever it goes. We will send it and this Journal for 1867 for $3, the price of the Watchman and Reflector alone, as above named. This offer will not be extended but withdrawn, not to be renewed, on the last day of 1866.

We should have been glad to have offered GODEY'S LADY'S BOOK of Philadelphia, with this Journal, at the price of Godey's alone, but they want to charge us as much as any other heathen man and publican, and we won't put up with it. It would have been different had Godey been at home, but he is abroad, and has left a representative behind him who has not a particle of common sense; we believe his name is Smith or Snooks, but he only controls the dollar and cent department. The soul of Godey is in Sarah J. Hale, and consequently whoever has three dollars to spare for a family periodical always welcomed by the girls, and always full of good things, useful and true, especially those taken from Hall's Journal of Health, would do well to invest it in Godey stock, sent to Louis A. Godey, Philadelphia, Penn., enclosing a Post Office Order for Three Dollars.

GARDENER'S MONTHLY, $2 a year, published by Brinceloe, 23 North 6th Street, Philadelphia, Penn., and by the Messrs. Woodward, 37 Park Row, New York.

This valuable and much prized Monthly is devoted to Horticulture, Arboriculture, Botany and the Rural Arts. January commences a new Vol., and from the variety of topics treated and the practical ability of the editor, Thomas Meehan, it will be valuable not only to families in the country, but to every household which owns a garden spot or cultivates a few flowers and plants for the window sill, or the conservatory in cities.

The advertising pages are of special value to farmers, gardeners and nurserymen, in giving information as to where agricultural instruments, manures, plants, &c., may be purchased.

AMERICAN EDUCATIONAL SERIES, and ALMANAC for 1867, by Ivison, Phinney, Blakeman & Co., with Catalogue of books for sale by that House.

LIFE AND DEATH ETERNAL; being a refutation of the Doctrine of Annihilation, by Samuel C. Bartlett, D. D., Professor in the Chicago Theological Seminary; 390 pp., 12mo.

To that happy company who, having been brought up in the nurture and admonition of the Lord by conscientious, praying parents, have never a doubt or a fear of the great fundamental truths of Evangelical Religion, we give the advice not to read the book any more than the writings of Hume, or Voltaire, or Tom Paine; they are happy and satisfied in their faith, there let them remain; but to the unhappy doubters, who are without God and without hope in the world, hence are restless, unfixed, and constantly looking forward uneasily into the future beyond the grave--to such we say, by all means read the book and it will be pretty sure to convince you that man is immortal and may become an heir to a glorious and undying existance in the mansions of the Blessed, where GOD and Angels are.

A FAMILY TREASURE

is the monthly publication of the Rev. Dr. McKinney, of Pittsburgh, Penn., sent for $1.50 a year. It is one of the few, the very few Monthlies which is always religious, and is always on the safe side. The articles are for the family, and are always practical, truthful and instructive; its sentiments tend to purify, to elevate and instruct; and yet are interesting to all. No family of any Christian denomination need have any fears of a malign influence on young minds, following the reading of any page of this well edited monthly. It is published at too low a rate and merits a wide patronage. We will send it and HALL'S JOURNAL OF HEALTH for 1867 to any present subscribers of either, for $2, which is very little over the subscription price of one of them provided the amount is sent to "Hall's Journal of Health, No. 2 West 43d St., New York," previous to the 5th day of December, 1866, when it will be withdrawn. This short offer is made in order to break up that miserable habit of putting off from day to day subscribing for a publication, for an indefinite period.

INVALIDS GOING SOUTH.

"AIKIN HOTEL" having been recently renovated and refurnished, is now open for the reception of visitors. Guests can rely on every exertion being made to render them comfortable and make them feel at home. The elevated situation of Aikin, with its dry, equable and genial climate, is peculiarly adapted to invalids affected with pulmonary diseases, and is highly recommended by eminent physicians, North and South.

HENRY SMEYSER, Proprietor.

AIKIN, South Carolina, Dec. 1, 1866.

---

☞ Your subscription ends with this number.

